# Chromosomal and mitochondrial diversity in *Melitaea didyma* complex (Lepidoptera, Nymphalidae): eleven deeply diverged DNA barcode groups in one non-monophyletic species?

**DOI:** 10.3897/CompCytogen.v10i4.11069

**Published:** 2016-12-06

**Authors:** Elena A. Pazhenkova, Vladimir A. Lukhtanov

**Affiliations:** 1 Department of Karyosystematics, Zoological Institute of Russian Academy of Sciences, Universitetskaya nab. 1, 199034 St. Petersburg, Russias; 2 Department of Entomology, St. Petersburg State University, Universitetskaya nab. 7/9, 199034 St. Petersburg, Russia

**Keywords:** Biodiversity, butterflies, *COI*, chromosome, karyotype, mitochondrial DNA, monophyly, non-monopyletic species, Nymphalidae, phylogeography, Pleistocene refugium, taxonomy

## Abstract

It is generally accepted that cases of species’ polyphyly in *COI* trees arising as a result of deep intraspecific divergence are negligible, and the detected cases reflect misidentifications or/and methodological errors. Here we studied the problem of species’ non-monophyly through chromosomal and molecular analysis of butterfly taxa close to *Melitaea didyma* (Esper, 1779) (Lepidoptera, Nymphalidae). We found absence or low interspecific chromosome number variation and presence of intraspecific variation, therefore we conclude that in this group, chromosome numbers have relatively low value as taxonomic markers. Despite low karyotype variability, the group was found to have unexpectedly high mitochondrial haplotype diversity. These haplotypes were clustered in 23 highly diverged haplogroups. Twelve of these haplogroups are associated with nine traditionally recognized and morphologically distinct species *Melitaea chitralensis* Moore, 1901, *Melitaea deserticola* Oberthür, 1909, *Melitaea didymoides* Eversmann, 1847, *Melitaea gina* Higgins, 1941, *Melitaea interrupta* Colenati, 1846, *Melitaea latonigena* Eversmann, 1847, *Melitaea mixta* Evans, 1912, *Melitaea saxatilis* Christoph, 1873 and *Melitaea sutschana* Staudinger, 1892. The rest of the haplogroups (11 lineages) belong to a well-known west-palaearctic species *Melitaea didyma*. The last species is particularly unusual in the haplotypes we obtained. First, it is clearly polyphyletic with respect to *COI* gene. Second, the differentiation in *COI* gene between these mostly allopatric (but in few cases sympatric) eleven lineages is extremely high (up to 7.4%), i.e. much deeper than the “standard” DNA barcode species threshold (2.7–3%). This level of divergence normally could correspond not even to different species, but to different genera. Despite this divergence, the bearers of these haplogroups were found to be morphologically indistinguishable and, most importantly, to share absolutely the same ecological niches, i.e. demonstrating the pattern which is hardly compatible with hypothesis of multiple cryptic species. Most likely such a profound irregularity in barcodes is caused by reasons other than speciation and represents an extraordinary example of intra-species barcode variability. Given the deep level of genetic differentiation between the lineages, we assume that there was a long period (up to 5.0 My) of allopatric differentiation when the lineages were separated by geographic or/and ecological barriers and evolved in late Pliocene and Pleistocene refugia of north Africa, the Iberian and Balkan Peninsulas, the Middle East and Central Asia. We discuss the refugia-within-refugia concept as a mechanism explaining the presence of additional diverged minor haplogroups within the areas of the major haplogroups. We also provide the first record of *Melitaea gina* in Azerbaijan and the record of *Melitaea didyma turkestanica* as a new taxon for Russia and Europe.

## Introduction

The *Melitaea didyma* (Esper, 1779) species complex, a group of taxa close to *Melitaea didyma* (Bryk 1940, Higgins 1941, Kolesnichenko 1999, Kolesnichenko et al. 2011) is widely distributed in the Palaearctic region. This complex exhibits a high level of individual and seasonal variation, although distinction between described taxa and between different populations in wing pattern is often unclear (Higgins 1941, 1955, Lvovsky and Morgun 2007, Oorschot and Coutsis 2014). Simultaneously these butterflies are similar in male and female genitalia structure (Higgins 1941).

The significant reviews of this complex were published by Bryk (1940), Higgins (1941, 1955), Kolesnichenko (Kolesnichenko 1999, Kolesnichenko et al. 2011), Tuzov and Churkin (2000). More recently the whole genus *Melitaea* Fabricius, 1807 was revised by Oorschot and Coutsis (2014). However, available cytogenetic (Lukhtanov and Kuznetsova 1989), morphological (Lvovsky and Morgun 2007, Kolesnichenko et al. 2011, Oorschot and Coutsis 2014) and molecular (Wahlberg and Zimmermann 2000, Lukhtanov et al. 2009, Dincă et al. 2015) data show that the *Melitaea didyma* species complex requires a more detailed study.

Combination of molecular and cytogenetic methods is a useful tool for detecting cryptic species (Lukhtanov et al. 2015) and can be a good addition to morphological analysis for ordering complex taxonomic structures (Lukhtanov et al. 2016). In our previous paper we applied analysis of DNA barcodes to demonstrate that *Melitaea didyma* complex is a monophyletic group and is represented by multiple deeply diverged mitochondrial DNA haplogroups (Pazhenkova et al. 2015).

In the present study we use a combination of molecular and chromosomal markers to analyse additional material collected in Armenia, Bulgaria, Georgia, Greece, Iran, Israel, Kazakhstan, Kyrgyzstan, Russia, Slovenia, Syria and Turkey, in order to reveal taxonomic and phylogeographic structure within the *Melitaea didyma* species complex. In our opinion, this group includes the following species: *Melitaea didyma* Esper, 1779, *Melitaea chitralensis* Moore, 1901, *Melitaea deserticola* Oberthür, 1909, *Melitaea didymoides* Eversmann, 1847, *Melitaea gina* Higgins, 1941, *Melitaea interrupta* Colenati, 1846, *Melitaea latonigena* Eversmann, 1847, *Melitaea mixta* Evans, 1912, *Melitaea saxatilis* Christoph, 1873 and *Melitaea sutschana* Staudinger, 1892. This complex does not include the taxa of the *Melitaea persea* complex (*Melitaea persea* Kollar, 1849, *Melitaea casta* Kollar, 1849, *Melitaea eberti* Koçak, 1980 and *Melitaea higginsi* Sakai, 1978) and the taxa of the *Melitaea ala* complex (*Melitaea ala* Staudinger, 1881, *Melitaea bundeli* Kolesnichenko, 1999, *Melitaea kotshubeji* Sheljuzhko, 1929, *Melitaea acraeina* Staudinger, 1886, *Melitaea enarea* Frühstorfer, 1917, *Melitaea ninae* Sheljuzhko, 1935 and *Melitaea didymina* Staudinger, 1895) which were shown to be strongly diverged with respect to genitalia structure (Higgins 1941, Kolesnichenko 1999, Oorschot and Coutsis 2014) and molecular markers (Leneveu et al. 2009).

## Material and methods

We studied standard *COI* barcodes (658-bp 5’ segment of mitochondrial *cytochrome oxidase subunit I*). We obtained *COI* sequences from 121 specimens collected in Armenia, Bulgaria, Georgia, Greece, Iran, Israel, Kazakhstan, Kyrgyzstan, Russia, Slovenia, Syria and Turkey. DNA was extracted from a single leg removed from each voucher specimen.

Legs from 21 specimens were processed at Department of Karyosystematics of Zoological Institute of the Russian Academy of Sciences. Primers and PCR protocol are given in our previous publications (Lukhtanov et al. 2014, Pazhenkova et al. 2015). Sequencing of double-stranded product was carried out at the Research Resource Center for Molecular and Cell Technologies of St. Petersburg State University. Legs from 100 specimens of *Melitaea* were processed at the Canadian Centre for DNA Barcoding (CCDB, Biodiversity Institute of Ontario, University of Guelph) using their standard high-throughput protocol described by deWaard et al. (2008). The set of voucher specimens of butterflies is kept in the Zoological Institute of the Russian Academy of Science (St. Petersburg).

The analysis involved 265 *COI* sequences (including outgroup) (Suppl. material 1). Among them there were 144 published sequences (Wahlberg and Zimmermann 2000, Vila and Bjorklund 2004, Leneveu et al. 2009, Lukhtanov et al. 2009, Dincă 2011, 2015, Hausmann et al. 2011, Ashfaq et al. 2013, Pazhenkova et al. 2015) collected from GenBank.

Within the studied samples, we are not completely sure of the identity of *Melitaea chitralensis* specimens (their barcodes were obtained from GenBank) because we were not able to check these vouchers and used the identification of these samples accepted in Ashfaq et al. (2013). According to Kolesnichenko (1999), *Melitaea chitralensis* is a member of the *Melitaea ala* subgroup, but the analysed samples clearly clustered with *Melitaea mixta*. Therefore, we can not exclude the possibility that these samples represent a north Pakistani population close to *Melitaea mixta*, but not a true *Melitaea chitralensis*.

Sequences were aligned using BioEdit software (Hall 1999). Mean uncorrected p-distances between haplogroups were calculated in MEGA7 (Kumar et al. 2015). Phylogenetic hypotheses were inferred using Bayesian inference (BI) as described previously (Vershinina and Lukhtanov 2010, Talavera et al. 2013a,b). Briefly, Bayesian analyses were performed using the program MrBayes 3.1.2 (Huelsenbeck and Ronquist 2001) with default settings as suggested by Mesquite (Maddison and Maddison 2015): burn-in=0.25, nst=6 (GTR + I +G). Two runs of 10 000 000 generations with four chains (one cold and three heated) were performed. Chains were sampled every 10000 generations.

Karyotypes were obtained from fresh adult males and processed as previously described (Vershinina et al. 2015). Briefly, gonads were removed from abdomen and placed to freshly prepared fixative (3:1; 96% ethanol and glacial acetic acid) directly after capturing butterfly in the field. Testes were stored in the fixative for 1 month at +4°C. Then the gonads were stained in 2% acetic orcein for 7-10 days at +18-20°C. Haploid chromosome numbers (n) were counted in meiotic metaphase I (MI) and metaphase II (MII).

## Results

### Karyotype

**Figure 1.**
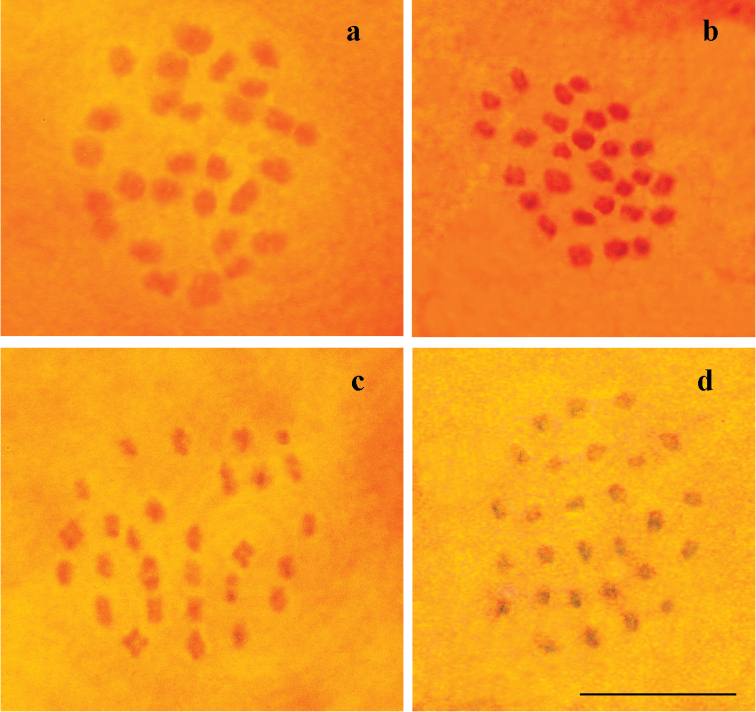
Karyotypes in male meiosis of *Melitaea gina* from Iran. **a** sample Q183, prometaphase I, n = 28 **b** sample Q153, late prometaphase I, n = 28 **c** sample Q183, MI, n = 28 **d** sample Q155, M I, n = 28. Scale bar corresponds to 10µ in all figures.

The haploid chromosome number n=28 was found in prometaphase I, MI and MII cells of seven studied individuals (Table 1, Fig. 1). All chromosome elements formed a gradient size row. The karyotype contained no exceptionally large or small chromosomes.

**Table 1.** Chromosome number and localities of *Melitaea gina* samples collected in Iran (province West Azerbaijan) (Collectors: V. Lukhtanov, E. Pazhenkova and N. Shapoval).

Sample	Karyotype	Haplotype	Locality	Altitude	Date
Q153	n=28	M18	25 km E of Mahabad (vic. Darman): N36°45'00"; E45°51'37"	1900–2000 m	10 August 2016
Q155	n=28		25 km E of Mahabad (vic. Darman): N36°45'00"; E45°51'37"	1900–2000 m	10 August 2016
Q156	n=28	M14	25 km E of Mahabad (vic. Darman): N36°45'00"; E45°51'37"	1900–2000 m	10 August 2016
Q157	n=28	M15	25 km E of Mahabad (vic. Darman): N36°45'00,30"; E45°51'36,60"	1900–2000 m	10 August 2016
Q182	n=28		25 km E of Mahabad (vic. Darman): N36°45'00"; E45°51'37"	1900–2000 m	10 August 2016
Q183	n=28		25 km E of Mahabad (vic. Darman): N36°45'00"; E45°51'37"	1900–2000 m	10 August 2016
Q211	n=28		3 km W of Khalifen: N36°44'35"; E45°32'13"	2100–2200 m	11 August 2016

### COI haplotypes and haplogroups

**Figure 2.**
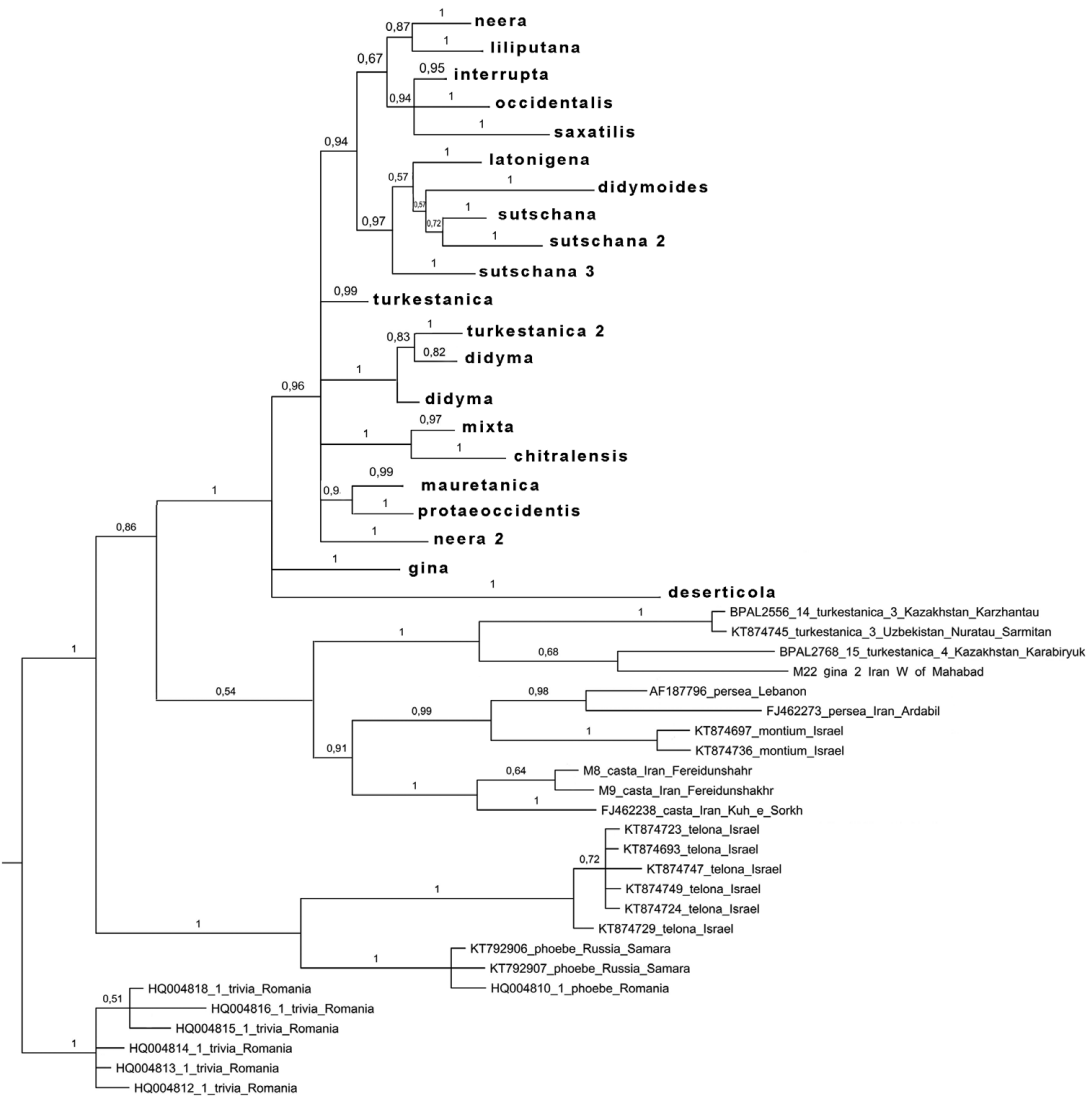
The Bayesian tree of *Melitaea* based on analysis of *the cytochrome oxidase subunit I* (*COI*) gene. Numbers at nodes indicate Bayesian posterior probability.

**Figure 3.**
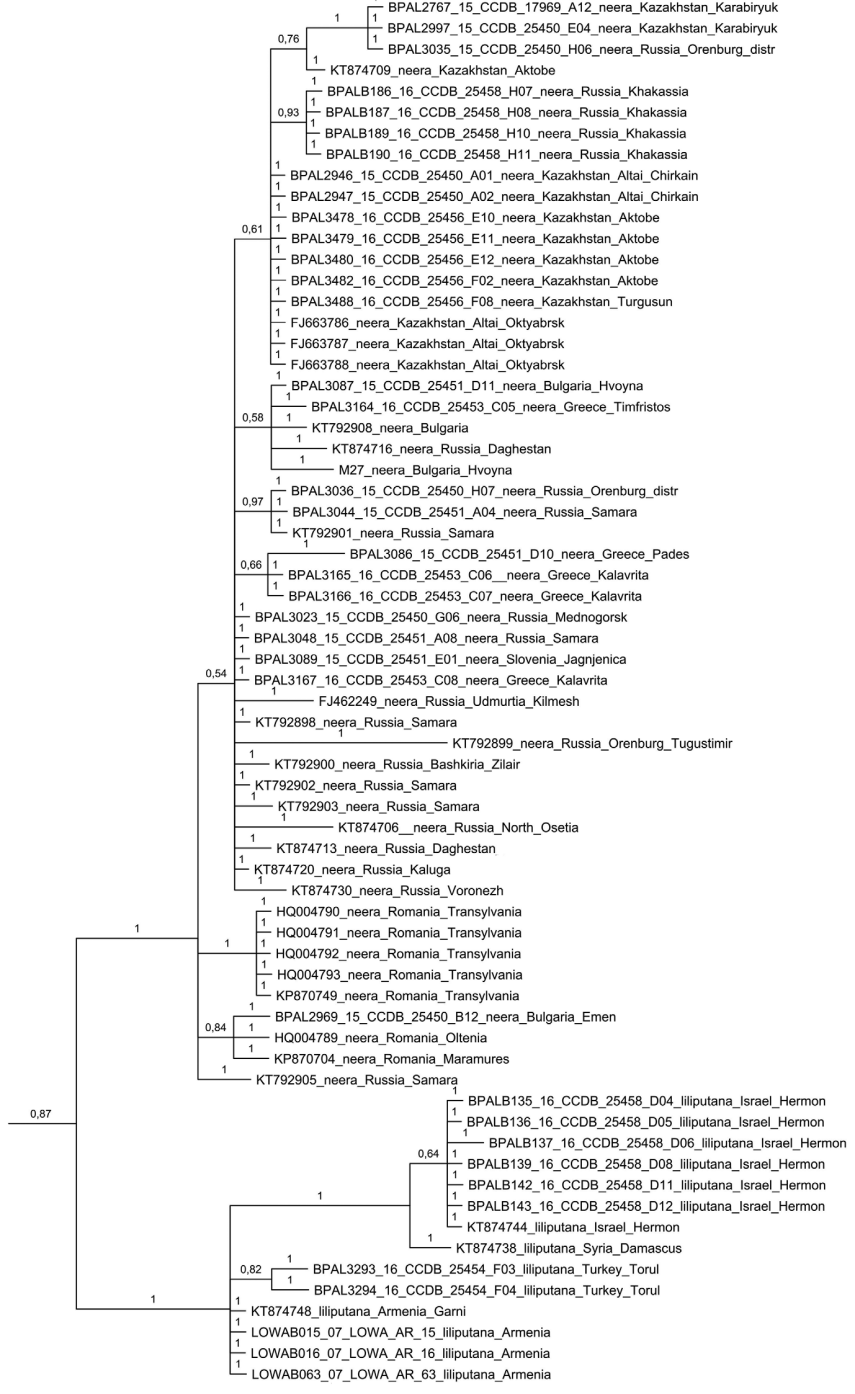
Fragment of the Bayesian tree of *Melitaea didyma* complex (haplogroups *neera* and *liliputana*) based on analysis of *COI* gene. Numbers at nodes indicate Bayesian posterior probability.

**Figure 4.**
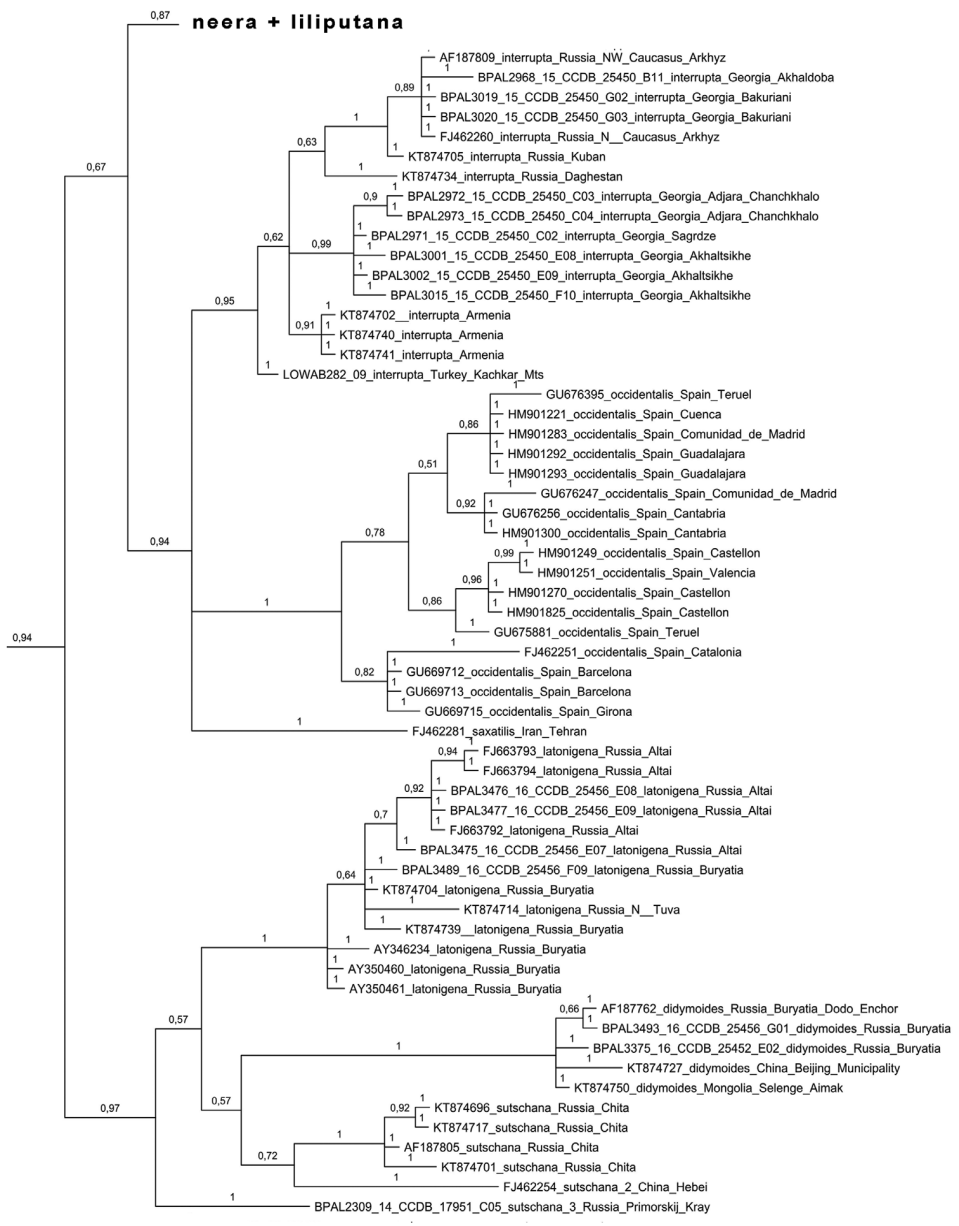
Fragment of the Bayesian tree of *Melitaea didyma* complex (haplogroups *interrupta*, *occidentalis*, *saxatilis*, *lathonigena*, *didymoides*, *sutschana*, *sutschana 2*, *sutschana 3*) based on analysis of *COI* gene. Numbers at nodes indicate Bayesian posterior probability.

**Figure 5.**
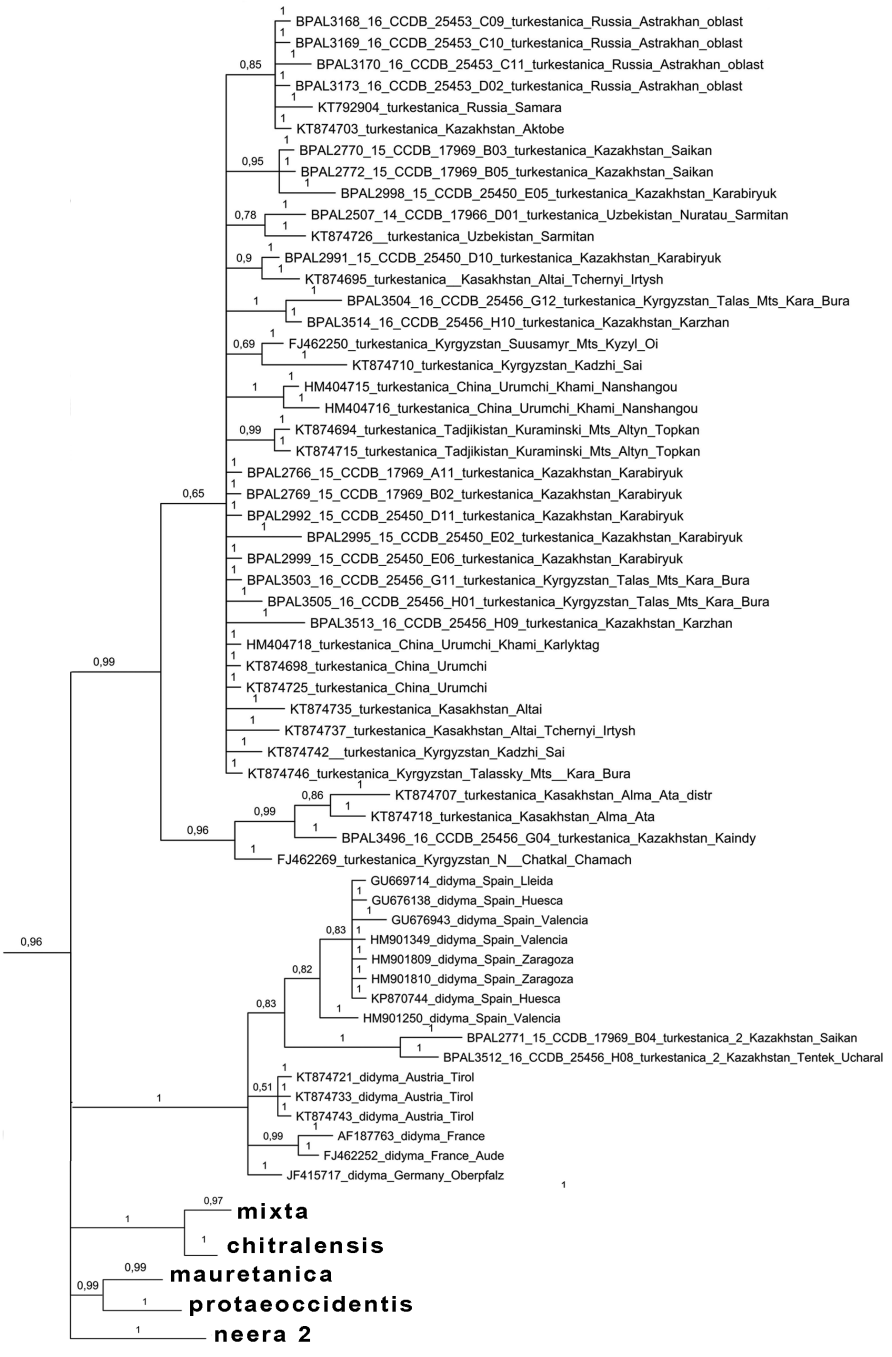
Fragment of the Bayesian tree of *Melitaea didyma* complex (haplogroups *turkestanica*, *turkestanica 2*, *didyma*) based on analysis of *COI* gene. Numbers at nodes indicate Bayesian posterior probability.

**Figure 6.**
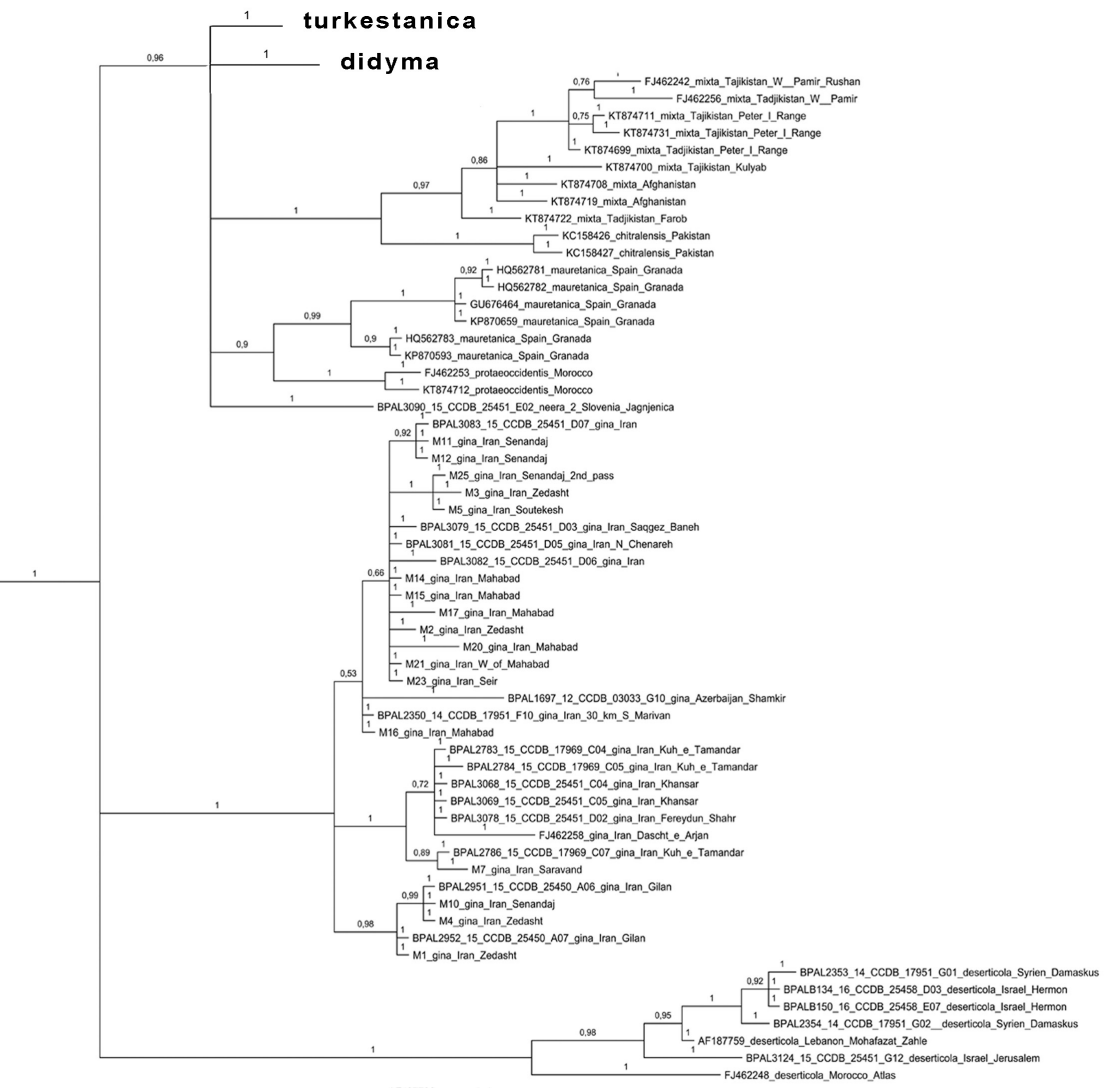
Fragment of the Bayesian tree of *Melitaea didyma* complex (haplogroups *mixta*, *chitralensis*, *mauretanica*, *protaeoccidentis*, *neera2*, *gina* and *deserticola*) based on analysis of the *COI* gene. Numbers at nodes indicate Bayesian posterior probability.

**Figure 7.**
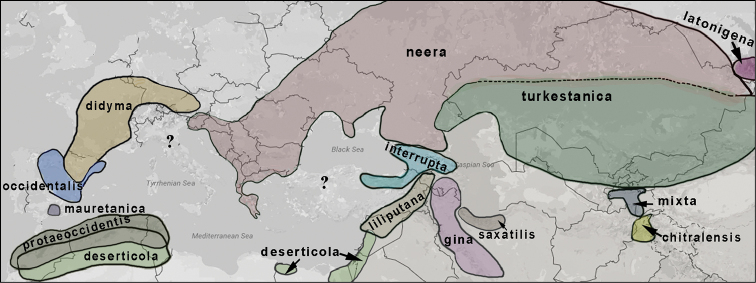
Distribution ranges of western *COI* haplogroups of *Melitaea didyma* complex.

Bayesian analysis of the barcode region recovered the *Melitaea didyma* complex as a monophyletic clade (Fig. 2), which agrees with Leneveu et al. (2009). Despite low karyotype variability, the clade was found to have unexpectedly high mitochondrial haplotype diversity. These haplotypes were clustered in 23 highly diverged haplogroups called *chitralensis*, *deserticola*, *didyma*, *didymoides*, *gina*, *gina2*, *interrupta*, *latonigena*, *liliputana*, *mauretanica*, *mixta*, *neera*, *neera2*, *occidentalis*, *protaeoccidentis*, *saxatilis*, *sutschana*, *sutschana2*, *sutschana3*, *turkestanica*, *turkestanica2*, *turkestanica3* and *turkestanica4* (Figs 2–6, Suppl. material 1). These haplogroups had high support (Bayesian posterior probability from 0.95 to 1) and were associated with particular geographical areas (Fig. 7).

The uncorrected mean *p*-distances between the haplogroups were high (up to 9.1% between *turkestanica4* and *deserticola*) (Table 2). The majority of them were much higher than the ‘standard’ 2.7–3.0% DNA barcode threshold usually used for allopatric taxa as an indicator for their species distinctness (Lambert et al. 2005, Lukhtanov et al. 2015).

**Table 2.** Mean uncorrected *COI* p-distances between 23 haplogroups of the *Melitaea didyma* species complex (%).

	1	2	3	4	5	6	7	8	9	10	11	12	13	14	15	16	17	18	19	20	21	22
1. chitralensis																						
2. deserticola	8.4																					
3. didyma	4.2	6.7																				
4. didymoides	6.3	6.9	4.7																			
5. gina	5.5	7.5	4.2	5.3																		
6. gina 2	6.4	9.5	6.5	7.2	6.5																	
7. interrupta	4.9	6.2	2.7	3.1	4.4	6.1																
8. latonigena	5.0	6.9	3.1	4.1	4.7	6.5	3.5															
9. liliputana	4.7	7.1	3.1	4.8	5.2	6.8	3.4	3.7														
10. mauretanica	4.1	6.3	2.1	4.2	4.8	7.1	2.2	3.1	3.5													
11. mixta	2.4	6.9	3.5	5.0	5.2	6.9	4.1	4.6	4.3	3.6												
12. neera	3.7	6.8	2.4	4.3	4.9	7.1	2.3	2.9	2.0	2.0	3.3											
13. neera 2	3.2	6.7	1.9	4.7	4.5	6.4	2.6	3.0	2.8	2.0	2.7	1.7										
14. occidentalis	4.9	6.9	2.9	3.9	4.6	6.4	1.8	3.8	3.9	2.4	4.0	2.8	2.4									
15. protaeoccidentis	3.3	5.6	2.1	4.1	4.2	6.7	2.7	2.7	3.0	2.1	2.9	2.1	2.0	3.0								
16. saxatilis	5.0	7.9	4.0	4.7	5.4	7.4	3.5	4.5	4.3	3.3	5.0	3.7	3.9	3.9	3.8							
17. sutschana	5.6	6.9	3.4	3.5	4.5	6.7	3.1	2.4	3.7	3.5	4.6	2.6	3.3	3.1	3.2	3.9						
18. sutschana 2	5.9	7.6	4.0	4.1	5.7	7.7	3.9	3.0	4.3	4.1	5.2	3.2	3.9	4.3	3.8	4.5	1.8					
19. sutschana 3	4.7	6.9	2.5	3.4	4.5	7.1	2.6	2.4	3.4	2.6	4.0	2.3	2.4	3.0	2.6	2.7	1.5	2.1				
20. turkestanica	3.4	7.0	2.3	4.4	4.3	7.0	3.0	3.4	3.1	2.4	2.7	2.1	1.6	3.1	2.3	3.7	3.6	4.3	2.8			
21. turkestanica 2	4.8	7.5	1.1	5.7	5.1	6.6	3.7	4.1	4.1	3.1	4.1	3.4	2.9	3.9	3.0	5.0	4.4	4.4	3.5	3.2		
22. turkestanica 3	7.0	8.9	5.8	8.9	6.4	6.4	4.8	6.1	7.1	7.0	6.3	7.9	6.9	5.8	6.0	6.2	7.0	6.9	7.0	6.6	6.4	
23. turkestanica 4	7.0	9.1	6.5	7.4	7.0	4.3	6.1	7.3	6.7	7.3	7.4	7.2	6.4	6.6	6.5	7.5	7.2	8.0	7.2	7.4	7.0	4.4

**Figure 8.**
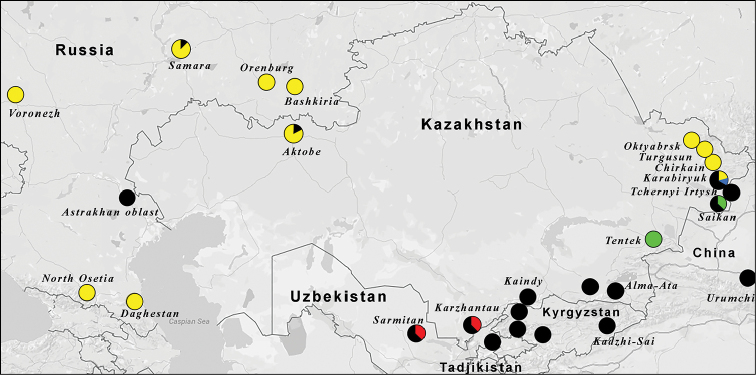
Localization of *neera* and *turkestanica* haplogroups (yellow circles – *neera*, black – *turkestanica*, green – *turkestanica2*, red – *turkestanica3*, blue – *turkestanica4*)

Most of the haplogroups were found to be allopatric. However, in some cases barcodes’ clusters did not correspond to the simple allopatric geographical distribution. The sample *Melitaea gina* M22 (haplogroup *gina2*) was found in sympatry with the haplogroup *gina* in north-west Iran. The distance between *gina* and *gina2* was 6.5%. Haplogroups *turkestanica4*, *turkestanica3* and *turkestanica2* were highly diverged (up to 7.4%) as compared with the haplogroup *turkestanica* and were found in sympatry with the haplogroup *turkestanica* (Fig. 8). In Slovenia, the specimen BPAL3090-15 (haplogroup *neera2*) was found together with the haplogroup *neera*. The distance between *neera* and *neera2* was 1.7%.

Two samples with the *turkestanica* haplotypes (haplogroup *turkestanica*), one from Aktobe (Kazakhstan) and one from Samara (Russia) were found in sympatry with *Melitaea dimyma neera* haplotypes (haplogroup *neera*). In Karabiryuk (Kazakhstan), two samples with the *neera* haplotypes (haplogroup *neera*) were found in sympatry with *Melitaea didyma turkestanica* haplotypes (haplogroup *turkestanica* and *turkestanica4*).

## Discussion

### Chromosome number variation

The genus *Melitaea* is known to be characterized by relatively low interspecific chromosome number variation. The representatives of basal clades (see phylogeny in Leneveu et al. 2009), the taxa of *Melitaea cinxia* (Linnaeus, 1758), *Melitaea diamina* (Lang, 1989), *Melitaea athalia* (Rottemburg, 1775), *Melitaea trivia* ([Denis & Schiffermüller], 1775) and *Melitaea phoebe* ([Denis & Schiffermüller], 1775) species groups demonstrate n=30-31 (Federley 1938, de Lesse 1960, Robinson 1971, Larsen 1975, Hesselbarth et al. 1995). These haploid numbers are modal ones not only for *Melitaea*, but also for the family Nymphalidae and for the order Lepidoptera in whole (Robinson 1971, Lukhtanov 2000, 2014). Most likely, one of them (probably, n=31, see Lukhtanov 2014) represents an ancestral lepidopteran condition preserved in the basal lineages of *Melitaea*.

The younger lineages, the *Melitaea fergana* Staudinger, 1882 and *Melitaea didyma* species groups, were found to possess lower chromosome numbers varying from n=27 to n=29-30. Within the *Melitaea fergana* species group, *Melitaea athene* Staudinger, 1881, the only karyologically studied species, was found to have n=29 (with n=30 as a rare intra-individual variation) (Lukhtanov and Kuznetsova 1989). The species-rich *Melitaea didyma* group consists of three complexes: a complex of taxa close to *Melitaea ala*, a complex of taxa close to *Melitaea persea* and a complex of taxa close to *Melitaea didyma*. Within these complexes the following chromosome numbers were found: n=29 in *Melitaea ala* (Lukhtanov and Kuznetsova 1989), n=27 in *Melitaea persea* (de Lesse 1960) and different numbers from n=27 to n=29-30 in species of the *Melitaea didyma* complex (Table 3).

**Table 3.** Chromosome numbers of taxa close to *Melitaea didyma*.

Taxon	Chromosome number	Country	Locality	Reference
*Melitaea didyma* ssp.	n=28	Italy	Abruzzi	de Lesse 1960
*Melitaea didyma neera*	n=28	Kazakhstan	Altai	Lukhtanov and Kuznetsova 1989
*Melitaea didyma neera*	n=27	Russia	N Caucasus, Pyatigorsk	Lukhtanov and Kuznetsova 1988
*Melitaea interrupta*	n=29	Turkey		de Lesse 1960
*Melitaea interrupta*	n=29	Azerbaijan, Nakhichevan	Zangezur Mts	Lukhtanov and Kuznetsova 1989
*Melitaea latonigena*	n=29–30	Kazakhstan	Altai	Lukhtanov and Kuznetsova 1989
*Melitaea deserticola*	n=29	Lebanon		Larsen 1975
*Melitaea gina*	n=28	Iran	W Azerbaijan	This study

**Note.** We did not include in the Table 3 the following data: *Melitaea* “*didyma*” (N Iran, Elburz, Demavend) n=28 (de Lesse 1960) because true *Melitaea didyma* is not known from Iran (van Oorschot and Coutsis 2014), and the studied samples could represent *Melitaea interrupta kendevana* or *Melitaea gina*. *Melitaea* “*didyma libanotica*” (Lebanon, Ain Zhalta Cedars) with n=27 (Larsen 1975) was also not included in the Table 2 since its identity remains unclear. The voucher samples for this count were larvae, and their identification was not certain. They actually could represent *Melitaea persea* (n=27 is typical number for *Melitaea persea*, including the population from Lebanon (de Lesse 1960).

Together with *Melitaea deserticola* (n=29, Larsen 1975), *Melitaea gina* occupies a basal position within the *Melitaea didyma* complex (Fig. 6). Therefore analysis of *Melitaea gina* was crucially important for understanding chromosome number evolution in this complex. Our study revealed *Melitaea gina* to have n=28, a number previously observed in *Melitaea didyma* from Italy (de Lesse 1960) and *Melitaea didyma neera* from the Kazakh Altai (Lukhtanov and Kuznetsova 1989). Taking into account absence or relatively low level of interspecific chromosome number variation in the *Melitaea didyma* complex and presence of intraspecific variation (Table 3), we conclude that in this group chromosome numbers have relatively low value as taxonomic markers (but see: Lukhtanov and Kuznetsova 1989).

### DNA barcode haplogroups and problem of non-monophyletic species

Despite low level of chromosome number variability, the *Melitaea didyma* complex was found to have unexpectedly high level of mitochondrial haplotype diversity. These haplotypes were clustered in 23 highly diverged haplogroups (Fig. 2). 12 of these haplogroups are associated with nine traditionally recognized and morphologically distinct species *Melitaea deserticola*, *Melitaea gina*, *Melitaea didymoides*, *Melitaea saxatilis*, *Melitaea sutschana* (this species was devided recently in *Melitaea sutschana* and *Melitaea yagakuana* Matsumura, 1927, see Oorschot and Coutsis 2014), *Melitaea latonigena* (this species was devided recently in *Melitaea latonigena* and *Melitaea latonigenides* Oorschot and Coutsis, 2014, see Oorschot and Coutsis 2014), *Melitaea interrupta*, *Melitaea mixta* and *Melitaea chitralensis*.

The rest of the haplogroups belong to the well-known west-palearctic species *Melitaea didyma*. Despite intrapopulation and seasonal variability, this species is very homogenous with respect to morphology, including the structure of genitalia, a character which is most useful for species separation in *Melitaea* (Suschkin 1913, Higgins 1941, Oorschot and Coutsis 2014). In accordance with this homogeinity, in the recent revision (Oorschot and Coutsis 2014) all populations of this species, except for Central Asian populations, were considered as members of the same subspecies *Melitaea didyma didyma*. The populations from Central Asia were treated by Oorschot and Coutsis (2014) as a separate subspecies *Melitaea didyma turkestanica*.

If we follow the opinion of experts in *Melitaea* taxonomy (Kolesnichenko et al. 2011, Oorschot and Coutsis 2014) and accept the traditional taxonomic treatment of the species *Melitaea didyma*, we should acknowledge that this species is particularly unusual in the haplotypes we obtained. First, it is clearly polyphyletic with respect to *COI* gene, and the lineages of *Melitaea didyma* are intermixed with other well recognized species on the tree (Figs 2–6). Second, the number of distinct *COI* lineages within *Melitaea didyma* is unusually high (11 lineages) and their genetic differentiation is extreme. The majority of these haplogroups are allopatric, but some of them have sympatric (*neera*/*neera1*, *turkestanica*/*turkestanica2*, *turkestanica*/*turkestanica3*, *turkestanica*/*turkestanica4*) or partially sympatric (*neera*/*turkestanica*, *occidentalis*/*didyma*) distribution. The mean uncorrected pairwise distances between the lineages is up to 7.4% if the lineages *turkestanica3* and *turkestanica4* are considered (Table 2). The lineages *turkestanica3* and *turkestanica4* are the most diverged lineages of *Melitaea didyma*. Together with *gina2*, on the tree (Fig. 2) they have an intermediate position between the lineage (*Melitaea didyma* + *Melitaea deserticola* + *Melitaea gina*) and the lineage (*Melitaea persea* + *Melitaea casta*). It even appears as a sister group to (*Melitaea persea* + *Melitaea casta*), but with a very low support (0.54). However, even if the lineages *gina2*, *turkestanica3* and *turkestanica4* are not considered, the distances between *Melitaea didyma* groupings remains high, up to 4.1% between *turkestanica2* and *liliputana*, i.e. much deeper than the “standard” DNA barcode species threshold (2.7-3%) (Hebert et al. 2003, Lukhtanov et al. 2016).

There are two theoretically possible explanations for this pattern. First, *Melitaea didyma* sensu auctorum can be a mix of multiple species that mostly have allopatric distribution ranges, but some of them are sympatric. Second, the recovered haplogroups (at least the allopatric ones) can represent highly diverged intraspecific lineages. Of course, a combination of the first and the second hypotheses is possible, and a part of the haplogroups could represent different species, and another part of the haplogroups could represent intraspecific variations.

In our opinion, the second hypothesis seems to be more plausible. There are the following arguments for the second scenario. First, no morphological differences between the bearers of these haplogroups are known (except for lighter, more yellowish wing colour in the three *Melitaea didyma turkestanica* haplogroups as compared with other haplogroups). The second (and the most convincing) argument is based on our field obseravtion of butterfly habitats and ecological preferences. In ecology the competitive exclusion principle, also known as Gause’s law is one of the most important rule (Gause 1934, Hardin 1960). In complete accordance with this rule, in case of sympatry the most closely related species pairs, such as *Melitaea didyma*/*Melitaea interrupta*, *Melitaea didyma*/*Melitaea latonigena* and *Melitaea gina*/*Melitaea saxatilis* demonstrate clear niche differentiation (*Melitaea didyma* and *Melitaea gina* are more xerophilous, whereas *Melitaea interrupta*, *Melitaea latonigena* and *Melitaea saxatilis* are more mesophilous taxa). This was not a case for sympatric haplogroups *neera*/*neera2*, *turkestanica*/*turkestanica*, *turkestanica*/*turkestanica3* and *turkestanica*/*turkestanica4* (Fig. 8). The bearers of these haplogroups were not only morphologically identical, but also were found to fly exactly syntopically and synchronously. This pattern is hardly compatible with non-conspecifity of these haplogroups.

*Melitaea didyma neera* and *Melitaea didyma turkestanica* are differentiated ecologically (Pazhenkova et al. 2015), however, there was no ecological separation between bearers of the *neera* and *turkestanica* haplogroups in cases of their sympatry. In Samara and Aktobe, where the haplogroup *neera* was predominant, both haplogroups were found in *Melitaea didyma neera* biotope (steppe), and in Karabiryuk where the haplogroup *turkestanica* was predominant, both haplogroups were found in *Melitaea didyma turkestanica* biotope (desert) (Fig. 8). This pattern corresponds more to a result of haplotype introgression than to co-habitation of two ecologically differentiated species.

Interestingly, the haplogroup *turkestanica2* is not related to the haplogroup *turkestanica* and is a derivative from West-European haplogroup *didyma*. This pattern can be treated as a result of ancient introgression. Generally, footprints of ancient and more recent introgression are both an evidence for transparency of boundaries between *Melitaea didyma* populations.

The mega-analysis of species-level para- and polyphyly in DNA barcode gene trees was recently conducted by using a huge data set (4977 species and 41,583 specimens of European Lepidoptera) (Mutanen et al. 2016), however without in-depth-analyses of particular cases. This study resulted in conclusion that cases of species’ polyphyly in *COI* trees arising as a result of deep intraspecific divergence were negligible, and the detected cases reflected misidentifications or/and methodological errors. Despite this, our analysis demonstrates that species-level polyphyly in DNA barcode based on deep intraspecific divergence may be a real phenomenon.

### Distribution ranges and phylogeography

The *Melitaea didyma* complex consists of at least 23 *COI* haplogroups, the majority of which demonstrated a strict attachment to particular geographic ranges: *chitralensis* (north Pakistan); *deserticola* (north Africa, Israel, Jordan, Lebanon, Syria); *didyma* (west Europe); *didymoides* (Asian Russia, Mongolia, North China); *gina* (W Iran, Azerbaijan); *interrupta* (Caucasus, NE Turkey); *latonigena* (Asian Russia, north-east Kazakhstan, Mongolia, north-west China); *liliputana* (Armenia, Turkey, Syria, Lebanon, Israel); *mauretanica* (south Spain); *mixta* (Tajikistan, Kyrgyzstan, Uzbekistan, Pakistan, Afghanistan); *neera* (east Europe, north Caucasus, west Siberia, north Kazakhstan); *occidentalis* (Spain); *protaeoccidentis* (north Africa); *saxatilis* (north Iran); *sutschana* (Russian Far East, Korea, north-east China) and *turkestanica* (Kazakhstan, Kyrgyzstan, Uzbekistan, Tajikistan, west China). With few exceptions (e.g. *deserticola*/*protaeoccidentis*, *deserticola*/*liliputana*), the ranges of these haplogroups do not overlap substantially (Fig. 7), and we hypothesize that mitochondrial diversity was formed in allopatry. Given the deep level of genetic differentiation between the lineages, we assume that there was a long period of allopatric differentiation when the lineages were separated by geographic or/and ecological barriers. Under generally accepted maximum 2.3% (Brower 1994) and minimum 1.5% uncorrected pairwise distance per million years (Quek et al. 2004) for *COI* sequence of various arthropod taxa, this period can be estimated to be as long as 0.5–5.0 My. In our opinion, this is an evidence that each of these haplogroups evolved in one of the main west-palaearctic late Pliocene and Pleistocene refugia in north Africa (*protaeoccidentis*, *deserticola*), the Iberian Peninsula (*occidentalis*, *mauretanica*), the Balkan Peninsula (*neera*), the Middle East (*liliputana*, *saxatilis*, *gina*) and Central Asia (*turkestanica*, *mixta*, *chitralensis*). The presence of additional diverged minor haplogroups *neera2*, *turkestanica2*, *turkestanica3*, *turkestanica4*, *gina2*, which could originate allopatrically in small isolated spots, but currently exist in secondary sympatry with major haplogroups *neera*, *turkestanica* and *gina*, agrees well with the refugia-within-refugia concept (Gòmez and Lunt 2007, Karaiskou et al. 2014). Interestingly, the area of the most diverged haplogroup *turkestanica3* is close to the area of the recently described subspecies *Melitaea didyma carminea* (Kolesnichenko et al. 2011).

### Taxonomic interpretation

We tentatively suggest interpreting the main clusters discovered within *Melitaea didyma* sensu stricto (*Melitaea didyma didyma*, *Melitaea didyma mauretanica*, *Melitaea didyma occidentalis*, *Melitaea didyma protaeoccidentis*, *Melitaea didyma liliputana*, *Melitaea didyma neera* and *Melitaea didyma turkestanica*) as subspecies because each of them has its own distribution range and is distinct with respect to mtDNA (i.e. represents by a monophyletic lineage or a combination of two or three monophyletic lineages). As a result we propose the following classification:

***Melitaea didyma* (Esper, [1779])**

***Melitaea didyma didyma* (Esper, [1779])**

***Melitaea didyma mauretanica* Oberthür, 1909**

***Melitaea didyma occidentalis* Staudinger, 1961**

***Melitaea didyma protaeoccidentis* Verity, 1929**

***Melitaea didyma liliputana* Oberthür, 1909**

***Melitaea didyma neera* Fischer de Waldheim, 1840**

***Melitaea didyma turkestanica* Sheljuzhko, 1929**

***Melitaea didymoides* Eversmann, 1847**

***Melitaea sutschana* Staudinger, 1892**

***Melitaea latonigena* Eversmann, 1847**

***Melitaea interrupta* Colenati, 1846**

***Melitaea mixta* Evans, 1912**

***Melitaea chitralensis* Moore, 1901**

***Melitaea deserticola* Oberthür, 1909**

***Melitaea saxatilis* Christoph, 1873**

***Melitaea gina* Higgins, 1941**

### New records

We provide the first record of *Melitaea gina* in Azerbaijan (sample BPAL1697-12, Azerbaijan, Shamkir, 27 June 2011, collector V. Tikhonov).

We also record *Melitaea didyma turkestanica* as a new taxon for Russia and Europe (samples BPAL3168-16, BPAL3169-16, BPAL3170-16, BPAL3173-16 Russia, Astrakhanskaya oblast, Bogdinsko-Baskunchaksky zapovednik, 24 May 2008, collector S. Nedoshivina).

## Supplementary materials

Supplementary material 1 Table S1 Data type: Microsoft Office Excel file Explanation note: List of *Melitaea* samples used in this study. This dataset is made available under the Open Database License (http://opendatacommons.org/licenses/odbl/1.0/). The Open Database License (ODbL) is a license agreement intended to allow users to freely share, modify, and use this Dataset while maintaining this same freedom for others, provided that the original source and author(s) are credited.Elena A. Pazhenkova, Vladimir A. Lukhtanov
